# Effects of dietary supplementation with different amounts of *Lycium ruthenicum* (black goji berry) branch roughage on plasma biochemical indices and rumen microflora of sheep

**DOI:** 10.3389/fmicb.2025.1556724

**Published:** 2025-04-08

**Authors:** Liangzhong Hou, Yan Ma, Jinlong Li, Yuxia Yang, Pingping Duan, Tongjun Guo

**Affiliations:** ^1^Feed Research Institute, Xinjiang Academy of Animal Sciences, Urumqi, China; ^2^Xinjiang Key Laboratory of Herbivorous Livestock Feed Biotechnology, Urumqi, China

**Keywords:** growth performance, rumen microbiota, sheep, *Lycium ruthenicum* branch roughage, rumen fermentation

## Abstract

This study aimed to evaluate the effects of dietary supplementation with roughage derived from black goji berry (*Lycium ruthenicum*) branches on plasma biochemical indices, and rumen microbiota in sheep. Forty male F1 lambs of Dupo×Hu sheep crossbreeds, approximately 4 months of age with similar body weight (29.58 ± 2.06 kg) and in good health, were selected and randomly divided into four groups (*n* = 10 per group). The control group was fed a full-mixed pelleted ration, and the experimental groups received the same diet supplemented with 10% (H1), 20% (H2) or 30% (H3) *L. ruthenicum* branch roughage. The addition of different amounts of *L. ruthenicum* roughage to the feed significantly increased the apparent digestibility of neutral detergent fiber, and antioxidant and immune indices of the sheep without negative effects on liver function. Additionally, the relative abundance of the gut bacteria NK4A214_group in the *Oscillospiraceae* family increased linearly and quadratically with the amount of *L. ruthenicum* roughage added to the diets. This findings suggest that bioactive components (e.g., flavonoids, terpenoids, organic acids) in *L. ruthenicum* branches may strengthen nutrient digestibility and immune performance by altering the type and abundance of rumen microbiota associated with fiber digestion and immunoregulation. Addition of 20–30% *L. ruthenicum* branch roughage to sheep fodder remarkably improved the growth performance and overall health.

## 1 Introduction

The rapid development of animal husbandry in recent years has exacerbated the problems related to forage availability, creating a serious imbalance between livestock feed requirements and available resources. This “grass-animal” contradiction, coupled with a reliance on limited roughage resources, constitutes a serious impediment to the continued development of the animal husbandry sector. The testing of non-conventional types of roughage is an important way to develop new sources (Martin et al., 2020). Increasing the proportion of non-conventional ground-sourced roughage in the feed can alleviate the shortage of plant resources and reduce feed costs. *Lycium ruthenicum,* commonly known as black goji berry is a thorny deciduous shrub in the *Solanaceae* family that has been used for centuries as a medicinal and food plant (Liu et al., 2024; Lu et al., 2021). It is believed to possess antioxidant, antitumor, hypolipidemic and immunity-enhancing effects (Islam et al., 2017). *L. ruthenicum* byproducts consisting of stalks, branches, leaves, unusable fruit, and processing waste contain significant amounts of proanthocyanidins, flavonoids, *Lycium barbarum* polysaccharides and alkaloids, and other polysaccharides, total flavonoids, amino acids and betaine (Dong et al., 2022), have the potential to be developed into beneficial feed additives (Xu et al., 2024). For example, in 2023 the area under cultivation for the related red golgi berry, *Lycium barbarum*, in China was about 160,000 mu (10,667 ha), and the annual output of by-products was 53,000 tons. The vast majority of black goji berry leaves as byproducts are burned causing local environmental degradation.

Some studies have shown that *L. ruthenicum* branches contain a large amount of crude protein, fiber and other nutrients, which can be turned into a dietary supplement that enhances the palatability of ruminants and improves production performance (Patra et al., 2022). Duan et al. increased final body weight, average daily gain, and the ability of Dupo×Hu sheep to digest crude protein by the addition of *L. ruthenicum* branches and leaves to the feed as roughage (Duan et al., 2024). The addition of *L. ruthenicum* pomace to feed rations of beach goats increased their dry matter intake, average daily weight gain, and rumen concentrations of acetic acid and propionic acid (Zhang et al., 2024). The underutilization of *L. ruthenicum* byproducts as animal feed supplements results in substantial waste. To effectively utilize the beneficial nutrients and bioactive compounds in these byproducts, it is imperative to determine the optimal inclusion rates in sheep diets to enhance feed utilization efficiency. Therefore, this investigation was designed to study the effects of the dietary addition of different percentages of *L. ruthenicum* branch roughage on the growth performance, antioxidant activity, immune system properties and rumen microbial diversity of sheep, with a view to providing an empirical basis for the utilization of *L. ruthenicum* branch roughage in sheep raising.

## 2 Materials and methods

### 2.1 Experimental location and ethical statement

The experiments were conducted from March 1 to May 9, 2023 at the sheep farm of Xinjiang Taihe Agriculture and Animal Husbandry Technology Co., Ltd., Bachu County, Kashgar Prefecture, Xinjiang (Kashgar, China). The study was carried out in accordance with the procedures sanctioned for this research, which were approved by the Science and Technology Ethics Committee of Xinjiang Academy of Animal Sciences, China (Approval No. 20230508). These procedures adhere to the principles and regulations for ethical protection in human and animal biological science and technology in China.

### 2.2 Experimental design

**Figure 1:**
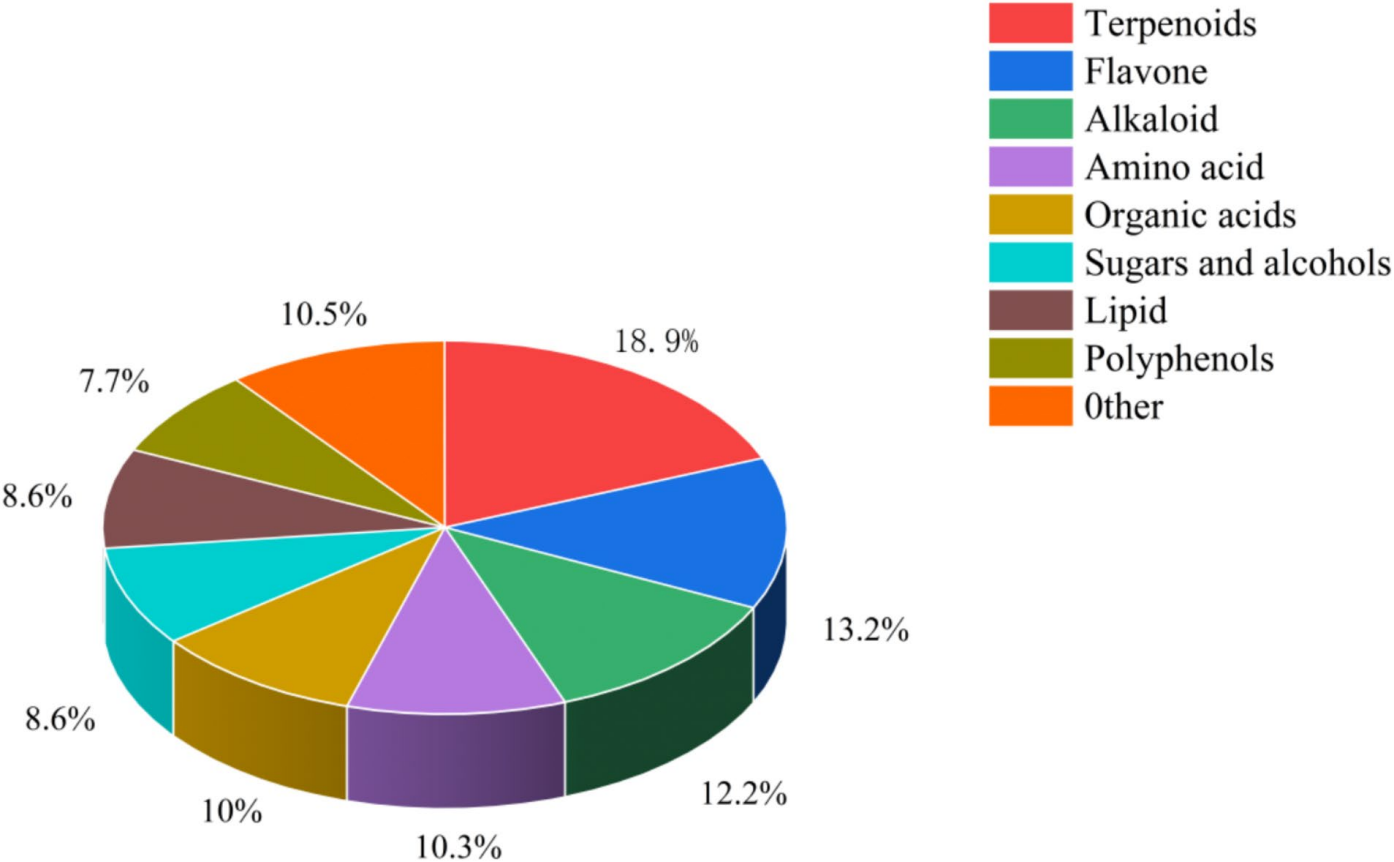
Classification of bioactive substances in the branches of *L. ruthenicum.*

Air-dried *L. ruthenicum* branches remaining after picking fresh fruit were provided by Xinjiang black fruit goji berry Biotechnology Company, Ltd., and their nutrient composition is shown in Table 1. The detection and quantitation of the bioactive ingredients in *L. ruthenicum* branch roughage was performed by the Baimaike Biological Company Ltd., using liquid chromatography-mass spectrometry (LC–MS) for detection. Figure 1 shows the results of the identification of the bioactive ingredients in *L. ruthenicum* branch roughage. The active components in the top nine categories of detected substances included terpenoids, flavonoids, alkaloids, amino acids, organic acids, sugars, alcohols, lipids and polyphenols. The active compounds in the top five categories of abundance were terpenoids (18.90%), flavonoids (13.20%), alkaloids (12.20%), organic acids (10.50%), and amino acids (10.30%). This study employed a completely randomized design to investigate the effects of incorporating *L. ruthenicum* branch roughage into the diet of lambs. Forty male lambs (4 months old, 30 ± 2 kg body weight) were dewormed and randomly assigned to four treatment groups (*n* = 10 replicates per group). The control group (CK) received a standard diet, while groups H1, H2 and H3 received the same diet supplemented with 10, 20 and 30% *L. ruthenicum* branch roughage, respectively (see Table 2 for the composition and nutrient analysis of the diet). The experimental period lasted 70 days, comprised of a ten-day acclimation period followed by a 60-day feeding trial.

**Table 1:** Nutrient content of *L. ruthenicum* branch roughage (as % dry matter, DM).

Item	DM	CP	EE	Ash	NDF	ADF	Ca	P
*L. ruthenicum* branch roughage	94.9	6.15	18.4	8.8	50.3	33.5	12.0	0.14

Nutrient contents are measured as % of DM, except for DM, which is measured on the basis of fresh weight. CP, crude protein; EE, ether extract; NDF, neutral detergent fiber; ADF, acid detergent fiber; Ca, calcium; P, phosphorus.

**Table 2:** Percent composition and nutrient contents of the basal diet (DM basis).

Items	Groups			
	CON	H1	H2	H3
Ingredients (%)				
Alfalfa hay	10.88	7.54	5.02	3.66
Soybean stalk	14.03	11.12	7.02	5.05
Straw	18.12	14.49	11.9	5.2
*L. ruthenicum* branch roughage	0	10	20	30
Corn	31.04	31.1	30.48	30.36
Wheat bran	7.89	7.74	7.67	7.72
Cottonseed meal	8.88	8.92	8.85	8.89
Sunflower meal	4.98	4.92	4.89	4.93
Limestone	0.12	0.12	0.11	0.13
NaCl	0.53	0.53	0.53	0.53
CaHPO_4_	0.51	0.5	0.51	0.51
NaHCO_3_	0.51	0.51	0.51	0.51
Premix^1)^	2.51	2.51	2.51	2.51
Total	100.00	100.00	100.00	100.00
Nutritional level				
ME, MJ/kg	11.06	11.21	11.69	11.66
CP, %	13.96	13.99	13.98	13.99
NDF, %	31.12	33.54	33.46	36.84
ADF, %	16.8	18.74	19.31	21.64
Ca, %	0.695	0.625	0.897	0.833
P, %	0.485	0.098	0.44	0.395

Each kg of premix contains Vit A ≥ 100,000 IU; Vit D3 ≥ 40,000 IU; Vit E ≥ 500 mg; Fe ≥ 2000 mg; Cu ≥ 280 mg; Zn ≥ 1,600 mg; Mn ≥ 800 mg; Se ≥ 75 mg; I ≥ 70 mg; Co ≥ 40 mg. CP, crude protein; NDF, neutral detergent fiber; ADF, acid detergent fiber; Ca, calcium; P, phosphorus.

### 2.3 Experimental design and animal feeding

Before the test, the sheep pens were cleaned and disinfected, and the test sheep were sheared, dewormed, and bathed. During the test period, the sheep were kept in separate pens and fed twice a day, at 10:00 a.m. and 18:00 p.m. The pens were equipped with a single water trough and a feed trough, and the sheep were free to feed and drink during the period.

### 2.4 Sample collection

#### 2.4.1 Feed and fecal samples

Feed samples were collected from each group every 7 days during the positive test period and stored in self-sealing bags at room temperature. At the end of the positive test period, the feed samples collected from each group were evenly mixed, and 200 g of feed samples were taken from each group by the quadratic method and stored in self-sealing bags for testing.

Fecal samples were collected by the whole-feces collection method. Five sheep were randomly selected from each group on the 57th day of the positive test period for the digestion and metabolism test, with an adaptation period of 7 days and a sampling period of 3 days. Fecal samples were collected every day before the morning feeding and weighed. The fecal samples collected over 3 days were mixed and 10% portions were removed and stored at 4°C for subsequent nutrient determinations.

#### 2.4.2 Determination of common nutrients

An analysis was conducted on samples of the feed, the ingredients, and the feces for DM (method 930.15), CP (method 990.03), EE (method 920.39), Ca (method 978.02), and P (method 946.06) using the AOAC procedures (Thanou et al., 2011). NDF and ADF content were determined by Van Soest’s method (Silva et al., 2011). ME was calculated from the measured nutritional value.


$$\begin{array}{l} \mathrm{Nutrient apparent digestibility}\;\left(\%\right)\\ =\left(\mathrm{dietary nutrient intake}-\mathrm{fecal nutrient excretion}\right)\\ /\mathrm{nutrient intake}\times 100\% \end{array}$$

#### 2.4.3 Plasma biochemistry, antioxidant and immune indices

On the 60th day of the positive test period, 10 mL blood samples were collected from the jugular vein into anticoagulant heparinized tubes, centrifuged at 4°C, 3000 g for 15 min, and the upper layer of plasma was removed and stored at −20°C. Glucose (GLU), creatine kinase (CK), urea nitrogen (BUN), total cholesterol (TC), triglycerides (TG), albumin (ALB), total protein (TP), high-density lipoproteins (HDL), low-density lipoproteins (LDL), glutamic oxaloacetic stress (AST), glutamic alanine aminotransferase (ALT), and lactic dehydrogenase (LDH) were determined with an AU480 automatic biochemical analyzer. Plasma IgA, IgG, IgM, malondialdehyde (MDA) concentration, superoxide dismutase (SOD), total antioxidant capacity (T-AOC) and glutathione peroxidase (GSH-Px) were measured by kits (Beijing Huaying Biotechnology Institute, Beijing, China).

#### 2.4.4 Rumen fluid sample collection and analysis

The rumen contents were collected and filtered through four layers of gauze. To avoid contamination, the first 50 mL of rumen fluid was discarded, and the pH of the remaining 30 mL was immediately measured using a portable pH meter (Shanghai Yidian Scientific Instrument Co., Ltd., Shanghai, China). A 10 mL aliquot of rumen fluid was stored in liquid nitrogen for subsequent analysis.

For determination of volatile fatty acids (VFAs) including acetic acid, propionic acid, butyric acid, isobutyric acid, valeric acid, and isovaleric acid, rumen fluid samples were thawed, and centrifuged at 3000 g for 10 min at 4°C. A volume of 0.4 mL was taken, mixed with 1.0 mL of distilled water and subjected to high-speed centrifugation. The supernatants were removed and VFA concentrations were determined using a Shimadzu GC2010 gas chromatograph equipped with a Stabilwax column.

Genomic DNA was extracted from samples using the TGuide S96 DNA kit (Tiangen Biotechnology Co., Ltd., Beijing, China) following the manufacturer’s instructions. The DNA concentration was measured using the Qubit dsDNA HS assay kit and Qubit 4.0 fluorometer (Invitrogen, Thermo Fisher Scientific, Oregon, United States), and quality was checked by 1% agarose gel electrophoresis. The V3–V4 region of the 16S rRNA gene was PCR amplified using a universal primer set:

338F: 5′- ACTCCTACGGGGAGGCAGCA-3′
806R: 5′-GGACTACHVGGGTWTCTAAT-3′

The PCR reactions contained KOD FX Neo 0.2 μL and ddH_2_O to 10 μL and VnF and Vn R were selected according to the amplification region. Thermocycler settings included an initial denaturation at 95°C for 5 min, followed by 25 cycles of denaturation at 95°C for 30 s, annealing at 50°C for 30 s, and extension at 72°C for 40 s. The final extension was at 72°C for 7 min. PCR amplicons were purified with Agencourt AMPure XP Beads (Beckman Coulter, Indianapolis, IN) and quantified with the Qubit dsDNA HS assay kit and Qubit 4.0 fluorometer (Invitrogen, Thermo Fisher Scientific, Oregon, United States). Libraries were constructed from equal amounts of pooled amplicons and sequencing was performed on an Illumina Novaseq 6,000 platform (Illumina, San Diego, California, US).

### 2.5 Statistical analysis of data

Data were organized using Excel 2016, and one-way analysis of variance (ANOVA) was performed using the ANOVA procedure of SPSS 26.0 statistical software. Multiple comparisons were performed using Duncan’s method if the differences were significant. The results were expressed as the mean and standard error of the mean (SEM) and regression analysis was done to test for linearity of the effects of increasing amounts of *L. ruthenicum* roughage added to feed. The differences were taken as significant at the *p* < 0.05 level and highly significant at *p* < 0.01.

## 3 Results and analysis

### 3.1 Apparent digestibility of nutrients

As can be seen from Table 3, with increasing amounts of *L. ruthenicum* roughage added to the feed, the apparent digestibility of NDF showed a tendency to increase and then decrease (*p* = 0.001). The H2 group was extremely or significantly higher than the CON and H3 groups, which increased by 10.13% (*p* < 0.01) and 15.16% (*p* < 0.05).

**Table 3:** Effect of *L. ruthenicum* roughage on the apparent digestibility of NDF in sheep.

Items	Groups				SEM	*p*-value		
	CON	H1	H2	H3		Total	Linear	Twice
DM	70.60	70.00	71.60	66.50	0.768	0.087	0.100	0.121
OM	59.76	59.64	59.41	56.06	0.650	0.123	0.049	0.193
CP	73.26	72.04	71.05	72.23	0.405	0.303	0.262	0.149
EE	55.67	55.84	57.63	55.57	0.661	0.684	0.810	0.428
NDF	43.72^Bbc^	44.76^ABb^	48.15^Aa^	41.81^Bc^	0.667	0.001	0.559	0.001
ADF	24.93	25.68	27.54	23.69	0.755	0.353	0.785	0.142
Ca	44.17	44.00	42.66	43.41	0.449	0.666	0.400	0.625
P	40.94	41.77	40.52	40.92	0.377	0.723	0.712	0.790

^A, B, C^ Different superscripts indicate significant differences within a row (*p* < 0.01). ^a, b, c^ Different superscripts indicate significant differences within a row (*p* < 0.05).

### 3.2 Biochemical indices in plasma

As can be seen from Table 4, increasing the amount of *L. ruthenicum* roughage added to the feed, resulted in a linear decrease in GLU content (*p* < 0.001), with each experimental group extremely significantly (*p* < 0.01) or significantly (*p* < 0.05) lower than that of the CON group, with decreases of 15.71, 26.33, and 22.48%, respectively. HDL-c showed a decreasing and then increasing trend, and each experimental group was extremely significantly (*p* < 0.01) or significantly (*p* < 0.05) lower than the CON group, with decreases of 26.23, 18.46, and 14.9%, respectively. The contents of IgA, IgG, IgM, T-AOC, SOD, and GSH-PX in each experimental group showed a linear or quadratic increase, and the concentration of MDA showed a linear or quadratic decrease (*p* < 0.01 or *p* < 0.05).

**Table 4:** Effect of dietary *L. ruthenicum* roughage on plasma indices of sheep.

Item	Group				SEM	*p-*value		
	CON	H1	H2	H3		Total	Linear	Twice
GLU, mmol/L	5.23^Aa^	4.52^Bb^	4.14^Bb^	4.27^Bb^	0.108	<0.001	<0.001	0.022
LDH, U/L	5261.65	5411.76	5257.28	4842.41	112.377	0.368	0.169	0.218
ALT, U/L	5.66	9.62	10.18	9.46	0.552	0.006	0.009	0.021
AST, U/L	20.55	31.89	32.65	29.38	1.524	0.008	0.031	0.021
CK, U/ml	0.26	0.36	0.28	0.22	0.016	0.008	0.113	0.004
TG, mmol/L	0.37	0.24	0.33	0.32	0.015	0.009	0.538	0.024
TC, mmol/L	1.81	1.76	1.84	2.03	0.038	0.063	0.023	0.095
HDL-c, mmol/L	0.77^Aa^	0.61^Bc^	0.65^Bbc^	0.67^Bb^	0.013	<0.001	0.061	<0.001
LDL-c, mmol/L	0.77	0.69	0.70	0.79	0.050	0.439	0.715	0.108
TP, g/L	75.95	75.22	74.03	73.55	0.887	0.777	0.312	0.945
ALB, g/L	29.73	31.5	30.12	30.66	0.312	0.222	0.610	0.324
BUN, mmol/L	9.21	8.39	8.57	8.91	0.159	0.217	0.624	0.074
IgA, g/L	0.73^Cc^	2.89^Bb^	2.8^Bb^	3.42^Aa^	0.184	<0.001	<0.001	<0.001
IgG, g/L	11.6^Bb^	11.61^Bb^	12.43^Bb^	19.7^Aa^	0.724	<0.001	<0.001	<0.001
IgM, g/L	1.29^Bb^	3.41^Aa^	3.34^Aa^	3.39^Aa^	0.166	<0.001	<0.001	<0.001
T-AOC, U/ml	6.85^Dd^	10.26^Aa^	8.47^Cc^	9.19^Bb^	0.227	<0.001	<0.001	<0.001
SOD, U/ml	59.35^Cc^	115.56^Aa^	88.89^Bb^	94.97^Bb^	3.681	<0.001	<0.001	<0.001
GSH-PX, U/ml	131.86^Cc^	217.39^Aa^	174.97^Bb^	186.1^Bb^	4.962	<0.001	<0.001	<0.001
MDA, nmol/ml	4.77^Aa^	2.22^Cc^	2.98^Bb^	3.34^Bb^	0.169	<0.001	<0.001	<0.001

^A, B, C^ Different superscripts indicate significant differences within a row (*p* < 0.01). ^a, b, c^ Different superscripts indicate significant differences within a row (*p* < 0.05).

### 3.3 Rumen fermentation parameters

The isobutyric acid content showed a linear decrease (*p* < 0.001) with increasing amounts of *L. ruthenicum* roughage added to the feed. The experimental groups were significantly lower than the CON group, with decreases of 21.05, 40.82, and 36.63%, respectively (Table 5).

**Table 5:** Effect of *L. ruthenicum* roughage on rumen fermentation of free fatty acids in sheep.

Item	Group				SEM	*p*-value		
	CON	H1	H2	H3		Total	Linear	Twice
pH	5.88	5.695	5.955	5.74	0.127	0.901	0.899	0.958
Acetate, mmol/L	107.33	89.04	94.45	94.90	3.156	0.216	0.251	0.138
Propionate, mmol/L	30.39	32.51	36.02	32.79	1.707	0.751	0.523	0.477
Iso-butyrate, mmol/L	1.38^a^	1.14^a^	0.98^b^	1.01^b^	0.056	0.026	0.007	0.145
Butyrate, mmol/L	26.66	24.17	25.25	23.77	1.563	0.933	0.629	0.885
Iso-valerate, mmol/L	2.28	2.13	1.691	1.89	0.140	0.500	0.232	0.552
Valerate, mmol/L	2.10	1.90	2.19	2.23	0.159	0.911	0.678	0.741
TVFAs, mmol/L	155.40	150.89	160.58	156.59	4.091	0.879	0.777	0.979
A/P, mmol/L	3.54	2.79	2.76	3.05	0.17	0.338	0.315	0.136

^A, B, C^ Different superscripts indicate significant differences within a row (*p* < 0.01). ^a, b, c^ Different superscripts indicate significant differences within a row (*p* < 0.05).

### 3.4 Ruminal microbiota

#### 3.4.1 Alpha diversity and OTU analyses

**Figure 2:**
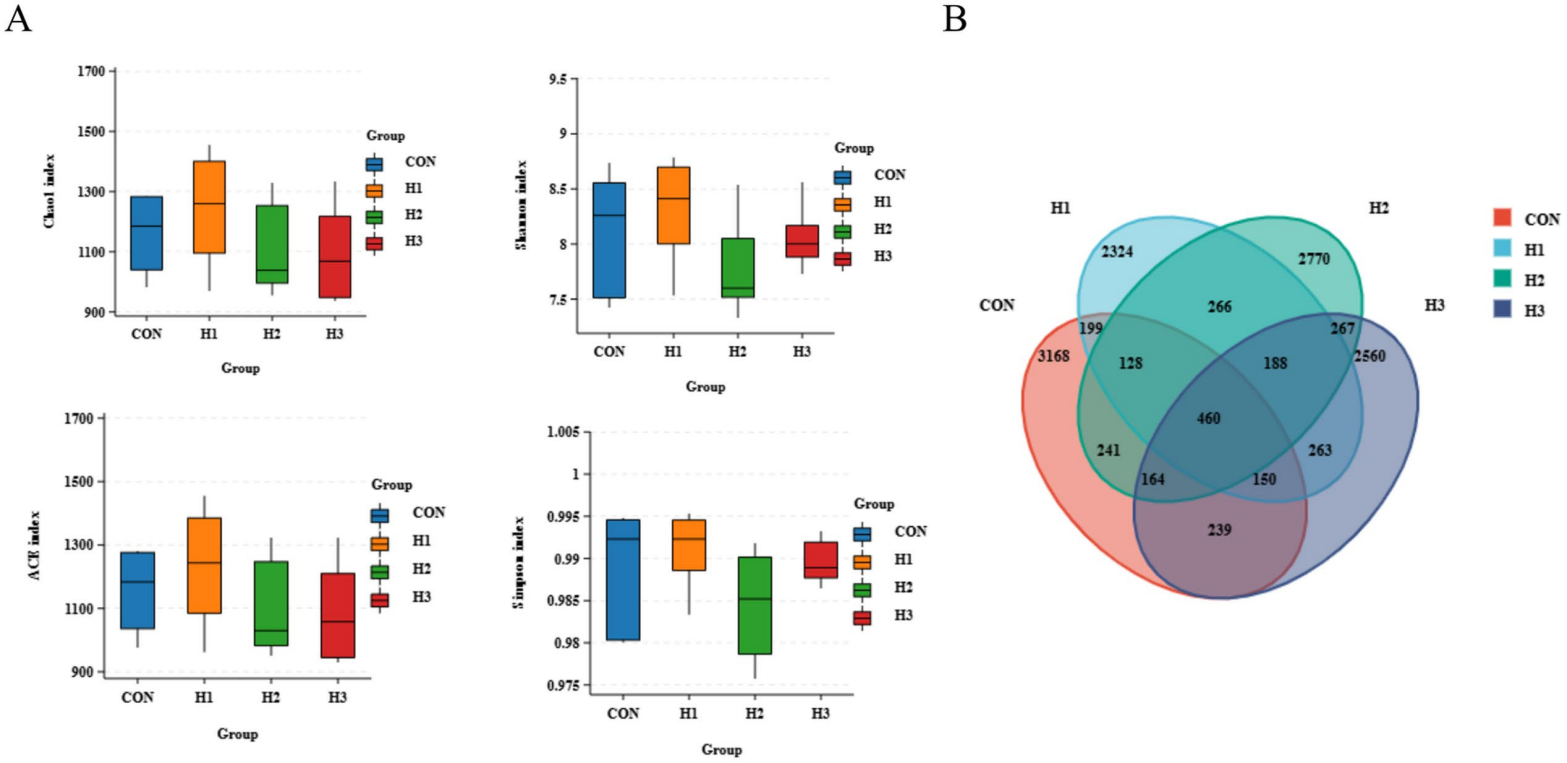
**(A)** Alpha diversity of ruminal microbiota in sheep. **(B)** Venn diagram of rumen fluid microbial overlap between the four groups.

The addition of different amounts of *L. ruthenicum* roughage to the feed had no significant effect on the alpha diversity of ruminal microbiota (*p* > 0.05) (Figure 2A). As shown in the statistics of OTUs in Figure 2B, sheep rumen fluid had a total of 460 microbial OTUs in the CON, H1, H2, and H3 groups. Among them, 3,168 OTUs were detected in the CON group, 2,324 OTUs in the H1 group, 2,770 OTUs in the H2 group, and 2,560 OTUs in H3 group.

#### 3.4.2 Species composition

**Figure 3:**
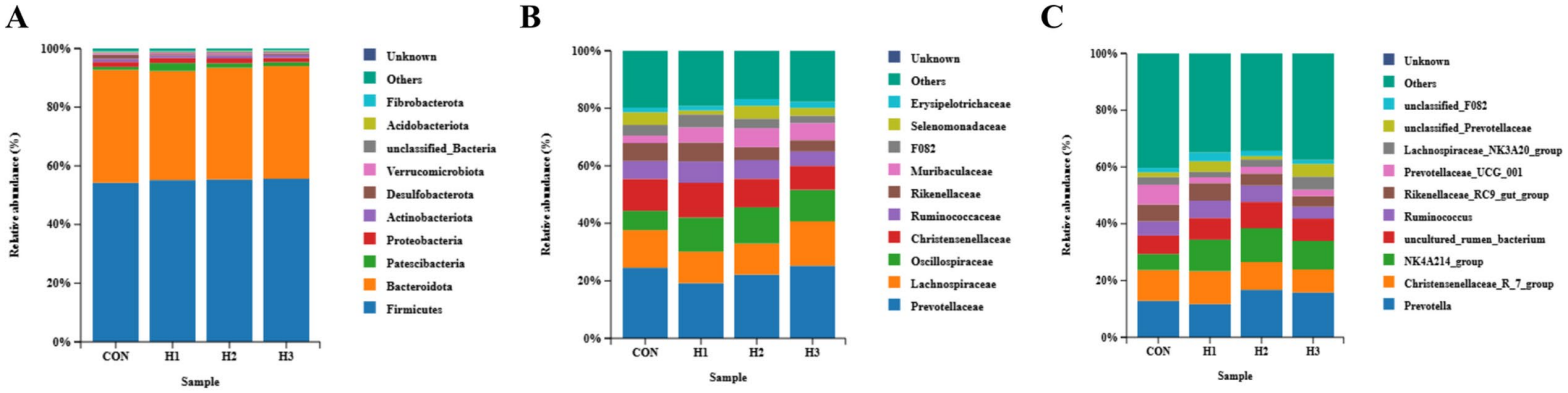
**(A)** Ruminal microbiota of sheep from each group at the phylum level, **(B)** the genus level, and **(C)** the species level.

As can be seen from Figure 3, the relative abundance of *Oscillospiraceae* showed a linear and quadratic increase (*p* < 0.05) with increasing levels of *L. ruthenicum* roughage addition to the diet (Figure 3B); the relative abundance of the NK4A214_group showed a trend of increasing and then decreasing (*p* < 0.05) with increasing levels of *L. ruthenicum* branches additions in the diet (Figure 3C); and the relative abundance of sheep ruminal microbiota phylum level species among groups had no significant effect (*p* > 0.05) (Figure 3A). There was no significant effect (*p* > 0.05) on the relative abundance of species at the level of ruminal microbiota phylum (Figure 3A).

#### 3.4.3 Linear discriminant analysis effect size (LEfSe) analysis

**Figure 4:**
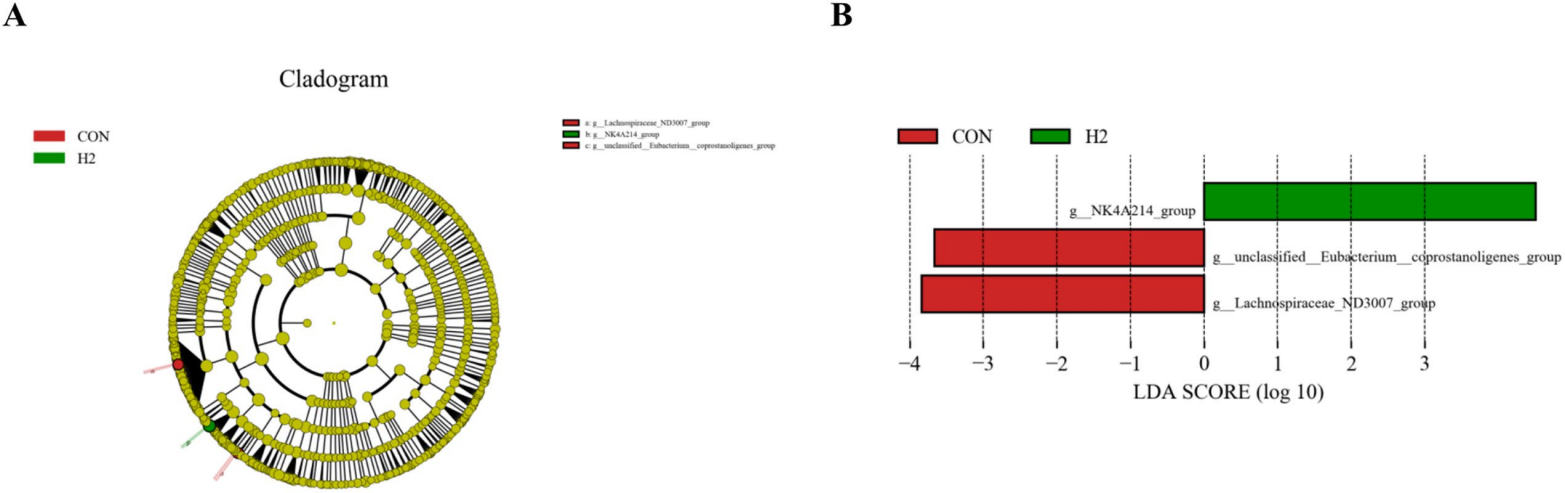
LEfSe analysis. **(A)** Branch maps showing the phylogenetic distribution of cecal bacteria in sheep fed a basal diet containing different amounts of *L. ruthenicum* branch roughage. **(B)** LDA scores showing the fraction of OTUs in cecal microbiota of sheep induced by feeding different amounts of *L. ruthenicum* branch roughage. Green, H2 group, 20% roughage; red, CON group, no roughage.

In Figure 4A, the microbial species with linear discriminant analysis (LDA) scores >4 are shown, and these represent microbial species with significant variability. In Figure 4B, the green bars show the greatest abundance of these bacteria in the H2 group, while the red bars show the greatest abundance of these bacteria in the CON group. There were two species with significance in the CON group, g_unclassified_Eubacterium_coprostanoligenes_group, and the g_ Lachnospiraceae_ND3007_group. There was one significant species in the H2 group, the g_NK4A214_group.

#### 3.4.4 Correlation analysis of differential rumen bacteria with volatile fatty acids, antioxidant and immune indicators

**Figure 5:**
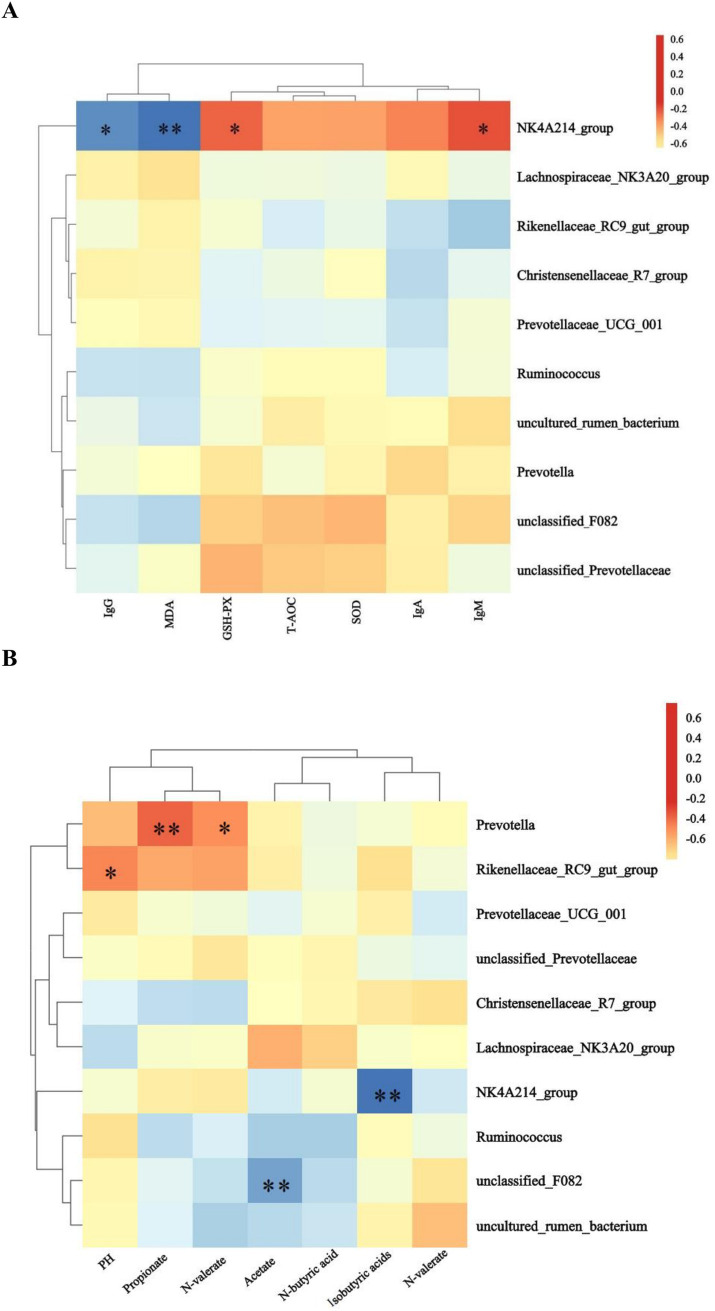
Heatmaps of the predominant rumen bacteria showing correlations with **(A)** antioxidants and immune indices and **(B)** volatile fatty acids.

The NK4A214_group showed significant (*p* < 0.05) or highly significant (*p* < 0.01) negative correlations with IgG and MDA, and significant positive correlations with GSH-PX and IgM (*p* < 0.05) (Figure 5A). The NK4A214_group showed significant (*p* < 0.05) or highly significant (*p* < 0.01) negative correlations with IgG and MDA and significant positive correlations with GSH-PX and IgM (*p* < 0.05) (Figure 5B). *Prevotella* showed highly significant (*p* < 0.01) or significant (*p* < 0.05) positive correlations with N-butyric acid and propionic acid, the *Rikenellaceae*_RC9_gut_group showed significant positive correlation (*p <* 0.05) with pH, the NK4A214_group showed a highly significant (*p* < 0.01) negative correlation with isovaleric acid, and unclassified_F082 showed a highly significant (*p* < 0.01) negative correlation with acetate.

## 4 Discussion

Measurement of biochemical parameters in the blood can indicate the nutritional status of sheep in a simple, quick way, which can help sheep farmers to avoid diseases caused by unbalanced nutrient intake, pinpoint any nutritional obstacles, and improve the economic benefits of sheep breeding. Serum ALT, AST, total TP and other tests reflect abnormal liver function, and serum BUN, TG and levels of certain metabolites are closely related to kidney function (Cai et al., 2021; Saemi et al., 2018). In this study, feed supplementation with increasing amounts of roughage derived from *L. ruthenicum* branches significantly increased plasma Glu, but had no significant effect on ALT or AST. This suggests that the amino acid composition of the feed was adequate, the protein content was high enough, and energy metabolism was sufficient. This diet meets the needs of the animals with respect to nutrient absorption and utilization with no adverse effects on liver metabolism.

Importantly, the HDL-c content of each experimental group decreased with increased dietary addition of *L. ruthenicum* branch roughage, indicating that the black goji berry branches contain terpenoids, flavonoids and other beneficial compounds that can reduce HDL cholesterol and prevent excess body fat deposition (Castellano et al., 2020; Wang et al., 2023). Free radical imbalance in the body causes oxidative stress, resulting in metabolic abnormalities and tissue damage (Li et al., 2022). Terpenoids and flavonoids are the main active immunomodulatory and antioxidant components in *L. ruthenicum* branches (Magalhães et al., 2022). In the present study, feed supplementation with increasing amounts of *L. ruthenicum* branch roughage highly significantly increased the antioxidant capacity of the sheep. This effect is likely mediated by terpenoid activation of the nuclear factor E2-related factor 2 (Nrf2)-heme oxygenase 1 (HO-1) pathway (Liang et al., 2021), leading to increased Nrf2 mRNA expression. Elevated Nrf2 levels subsequently could upregulate downstream antioxidant enzymes such as superoxide dismutase (SOD), catalase (CAT), and glutathione peroxidase (GSH-Px), while decreasing malondialdehyde (MDA) levels.

In addition, a linear increase in IgA, IgG, and IgM content was observed with increasing *L. ruthenicum* roughage supplementation. This finding aligns with Zhang et al. (2022) who demonstrated that fermented *Lycium barbarum* byproducts stimulated sheep immune responses by regulating purine and pyrimidine metabolism, thereby increasing IgG, IgA, and IgM levels, and enhancing the immunity of the animals, which was consistent with the results of the present study. This suggests that flavonoids in *L. ruthenicum* may interact with the Toll-like receptor 4 (TLR-4)-mediated intracellular signaling cascade, inhibiting the release of tumor necrosis factor-*α* (TNF-α), interleukin-6 (IL-6), and interleukin-1β (IL-1β) from macrophages. Consequently, the production and accumulation of nitric oxide was inhibited (Kim et al., 2020; Tang et al., 2011), promoting the release of immune factors. As the core mediators of the inflammatory cascade, a decrease in TNF-α and IL-1β concentration has been shown to effectively alleviate chronic low-grade inflammation, thus reducing the risk of metabolic abnormalities and tissue damage caused by systemic inflammation (Han et al., 2023). Moderate regulation of IL-6 plays a key role in maintaining immune homeostasis, and excessive inhibition may affect the acute immune response (Kerkis et al., 2024). It should be emphasised that the cytokine network is highly synergistic, TNF-α has the capacity to positively regulate IL-6 secretion, while anti-inflammatory factors such as IL-10 may participate in the regulation through a negative feedback mechanism (Kalb et al., 2021). Consequently, the regulation of the inflammatory network by *L. barbarum* branches may involve multi-factor interactions rather than a single pathway, a hypothesis that is supported by the linear increase trend of immunoglobulins such as IgA, IgG, and IgM. The moderate activation of the immune system, in conjunction with the selective inhibition of inflammatory factors, constitutes the optimal configuration of the body’s defence ability.

Alpha diversity analysis mainly reveals the abundance and microbial composition of the gut microbiome, as well as the coverage information of the sample microorganisms under this sequencing condition. In this study, a Venn diagram generated from the data and alpha diversity analysis based on OTU level showed no significant differences between groups, indicating that the addition of *L. ruthenicum* roughage to the diet had no adverse effect on the structure of the rumen microbiome in sheep. The ruminal microbiota are an important part of the intestinal digestive system, and at the same time, the structure and type of diet is one of the key factors affecting intestinal microecological balance (Tayyab et al., 2022). *Oscillospiraceae* is an important family in the rumen whose members produce fatty acids such as butyrate and propionate, increase cuprocytes and mucus, and maintain the integrity of the intestinal epithelial barrier, thus inhibiting the colonization of harmful intestinal microbes (Wang et al., 2024). The NK214_group belongs to the *Ruminalococcaceae* family of bacteria, some of whose members produce cellulase, which breaks down the cell walls in fibrous materials, thus increasing nutrient absorption from high-fiber feeds by ruminants (Niu et al., 2022; Ran et al., 2021). In this study the relative abundance of the NK4A214_group (*Oscillospiraceae*) was significantly increased and propionic acid content was slightly elevated. The NK4A214_group was negatively correlated with IgG, MDA, and isobutyric acid and positively correlated with GSH-PX and IgM. The study showed that the NK214_group was the predominant genus in the rumen microbiota, which could increase the apparent digestibility of ADF and NDF, consistent with our previous findings (Table 3), supporting the hypothesis that high content of NDF in the *L. ruthenicum* branches was the main source of the increase in the NK4A214_group, which degrades the crude fibers in the roughage and produces fatty acids that provide energy to the host and mediate the immune and inflammatory responses of the body (Li et al., 2024). Cheng et al. reported that green brick tea extract (QZT) was found to have significant anti-obesity, free radical scavenging, and antioxidant properties. QZT extract inhibited adipocyte proliferation, improved gut microbe-mediated metabolic disorders in high-fat mice and reduced *Oscillospira* abundance, which was associated with metabolic syndrome (Cheng et al., 2022). This suggests that sheep may benefit from increased abundance of *Oscillospira* to enhance energetic effects and thus antioxidant and immune properties. *Prevotella* is an abundant bacterial genus in the rumen, possessing the desirable functions of degrading cellulose, starch, hemicellulose and protein (Li et al., 2017) to produce propionic acid and N-valeric acid. The RC9_gut_group belongs to the *Rikenellaceae* family, which are the main rumen microorganisms that play an important role in fiber digestion by secreting a large number of cellulases and hemicellulases to degrade cellulose and hemicellulose in the feed (Huang et al., 2020; Zened et al., 2013). Therefore, we hypothesize that the bioactive components (flavonoids, terpenoids, organic acids, etc.) enriched in *L. ruthenicum* branch roughage could influence nutrient digestibility and immune performance by increasing the abundance of specific rumen microbes associated with fiber digestion and immunosuppression.

## 5 Conclusion

The results of this experiment bioactive components (e.g., flavonoids, terpenoids, organic acids) in *L. ruthenicum* branches may strengthen nutrient digestibility and immune performance by altering the type and abundance of rumen microbiota associated with fiber digestion and immunoregulation. Addition of 20–30% *L. ruthenicum* branch roughage to sheep fodder remarkably improved the growth performance and overall health.

## Data Availability

The raw data supporting the conclusions of this article will be made available by the authors, without undue reservation.
